# Promoting participation in remote digital health studies: An expert interview study

**DOI:** 10.1177/20552076231212063

**Published:** 2023-11-13

**Authors:** Paola Daniore, Vasileios Nittas, Felix Gille, Viktor von Wyl

**Affiliations:** 1 Institute for Implementation Science in Health Care, 27217 University of Zurich, Zurich, Switzerland; 2 Digital Society Initiative, 27217 University of Zurich, Zurich, Switzerland; 3 Department of Behavioral and Social Sciences, 6752 Brown University, Providence, USA; 4 Epidemiology, Biostatistics and Prevention Institute, 27217 University of Zurich, Zurich, Switzerland

**Keywords:** Digital health, health informatics, technology smartphone, social media, digital clinical trials, remote clinical trials

## Abstract

**Background:**

Remote digital health studies are on the rise and promise to reduce the operational inefficiencies of in-person research. However, they encounter specific challenges in maintaining participation (enrollment and retention) due to their exclusive reliance on technology across all study phases.

**Objective:**

The goal of this study was to collect experts’ opinions on how to facilitate participation in remote digital health studies.

**Method:**

We conducted 13 semi-structured interviews with principal investigators, researchers, and software developers who had recent experiences with remote digital health studies. Informed by the Unified Theory of Acceptance and Use of Technology (UTAUT) framework, we performed a thematic analysis and mapped various approaches to successful study participation.

**Results:**

Our analyses revealed four themes: (1) study planning to increase participation, where experts suggest that remote digital health studies should be planned based on adequate knowledge of what motivates, engages, and disengages a target population; (2) participant enrollment, highlighting that enrollment strategies should be selected carefully, attached to adequate support, and focused on inclusivity; (3) participant retention, with strategies that minimize the effort and complexity of study tasks and ensure that technology is adapted and responsive to participant needs, and (4) requirements for study planning focused on the development of relevant guidelines to foster participation in future studies.

**Conclusions:**

Our findings highlight the significant requirements for seamless technology and researcher involvement in enabling high remote digital health study participation. Future studies can benefit from collected experiences and the development of guidelines to inform planning that balances participant and scientific requirements.

## Introduction

Engaging participants remains a crucial challenge for longitudinal digital health studies, which use digital technologies to monitor participants over time for health outcomes.^1–3^ Studies often struggle to recruit and retain adequately large, diverse, and representative samples. This is often due to barriers, such as the time and financial resources, needed to get participants to enrollment locations, as well as the staffing resources required to plan and implement studies comprehensively.^4,5^ In turn, studies suffer from reduced external and internal validity, which limits their real-world application.^6^

A new wave of longitudinal digital health studies aims to overcome these challenges by shifting participation and all study phases entirely online, otherwise referred to as remote digital health studies.^7,8^ Remote digital health studies aim to reduce the operational inefficiencies of in-person research and provide a more personalized participant experience.^9,10^ Furthermore, they aim to make digital health studies more inclusive by leveraging widespread smartphone ownership and internet access. Through passive and scalable approaches to leverage these technologies, such as remote monitoring, remote digital health studies can particularly benefit those facing physical and social barriers like marginalized communities, people living with chronic diseases, and the elderly.^11^

Evidence suggests that remote digital health studies achieve greater participant retention than in-person digital health studies.^8,12,13^ Yet, enrollment of representative samples and participant attrition remain challenging.^14^ To overcome these challenges, remote digital health studies often rely on additional measures, such as the provision of financial incentives, motivational messages, and the adaption of digital health tools to offer a more personalized participant experience.^15–17^

However, strategies to increase participation often are merely based on the assumptions and preferences of the research groups.^8^ To date, there has been no scientific exploration of why and how these strategies are chosen, the experienced advantages or disadvantages of the chosen strategies, or how they impact participation success. At the same time, remote digital health studies are rapidly growing in popularity, with no guideline available to support study planning.^7^ This presents an opportunity for experts to align on best practices on how to increase participation and improve the scientific impact of remote digital health research. We aim to explore the following questions:

1. What are the motivators and barriers to participation in remote digital health studies?
2. Which additional measures should researchers or software developers take to increase participation in remote digital health studies?
3. How could a guideline best inform future remote digital health study planning from a participation-focused perspective?

To address these questions, we conducted qualitative expert interviews informed by a scoping review on participation in remote digital health studies^8^ and the Unified Theory of Acceptance and Use of Technology (UTAUT) framework.^18^ UTAUT provides a structure of experience and usability factors linked with digital technologies, which are used across all remote digital health study phases.^19,20^ Understanding whether participation strategies are aligned with participant requirements for their adoption is a first step in developing evidence-based guidance.

## Methods

### Study sample and design

Our sample included expert principal investigators, researchers, and software developers involved in developing and conducting remote digital health studies worldwide. We decided to also include software developers due to their central role in designing the digital tools used across all phases of digital health studies.^21^ Potentially eligible participants were identified from original research articles through a scoping review we recently conducted that assessed participation in remote digital health studies.^8^ We initially invited all potential participants via email, introducing them briefly to the research team and the study's aims. We then sent interested participants a second email, including informed consent and a more detailed study plan. Those who confirmed participation were asked to digitally sign and return the informed consent form before the arranged interview dates. Reminder emails were sent 1 week after the initial contact. Additional interviewees were recruited through snowball sampling, where the experts we interviewed referred us to other possible interviewees. These included experts, such as software developers not directly involved in the conduct of studies, who would have not appeared in our literature search.^22^ That led to a second round of interviews.

Semi-structured interviews were conducted and recorded via Zoom's video conferencing platform (version 5.7.7). We used an interview guide that was co-developed with all the authors. The development of the questions in the interview guide was informed by the constructs of a framework introduced in a scoping review on participation in remote digital health studies^8^ and the UTAUT framework^18^ (see Appendix 1). The scoping review presents three central constructs that affect participation in remote digital health studies, while the UTAUT framework presents four central constructs that affect individuals’ intention to adopt or use technologies.^23^ We specifically chose the UTAUT framework to guide the interviews as it assesses each factor across all technology-enabled phases of remote digital health studies through a participant's lens. This allows for the identification of additional influences that may affect their participation.

### Data collection and analysis

**Figure 1.**
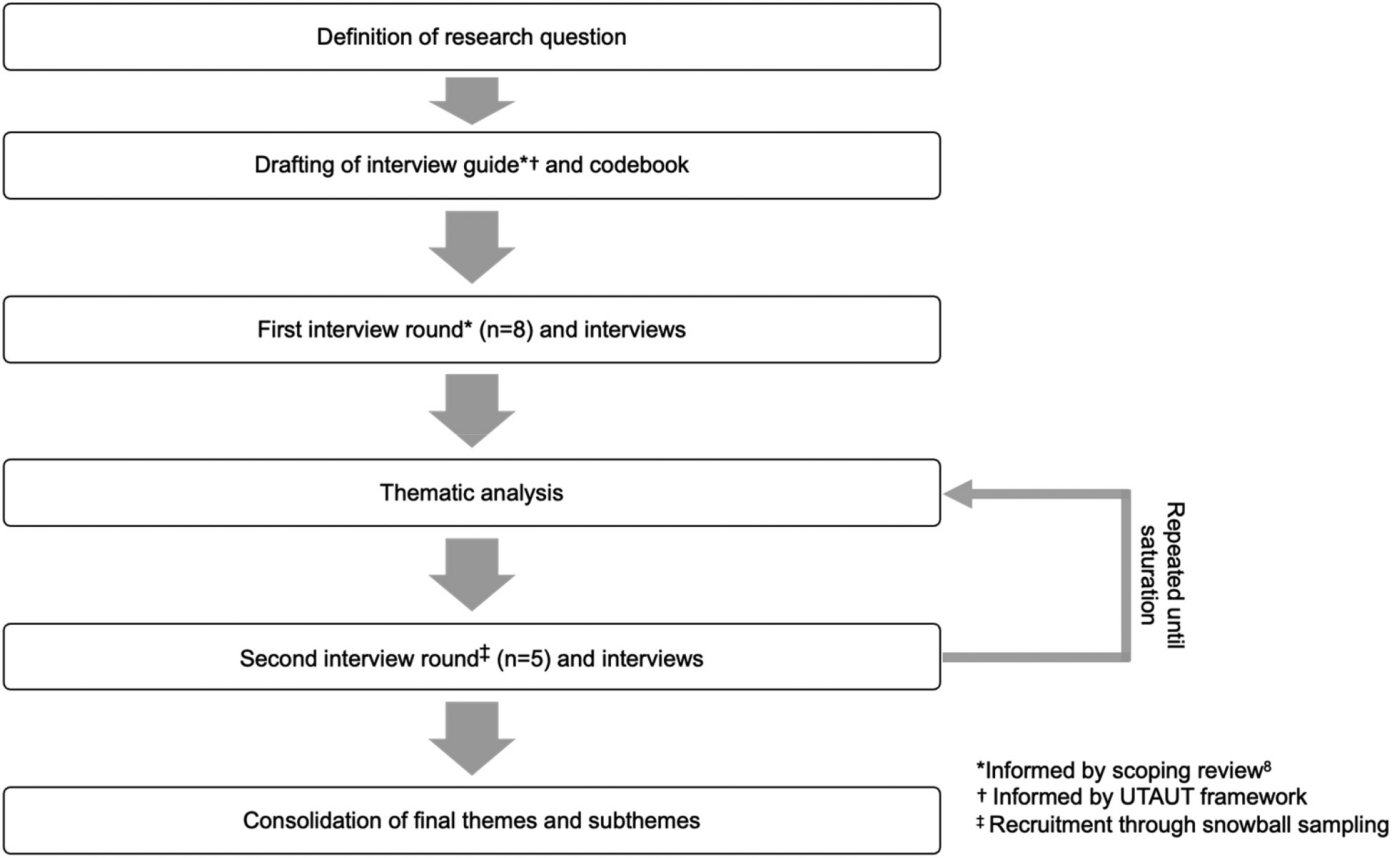
Flowchart of study methods.

Our study followed a thematic approach implemented over two interview rounds (Figure 1).^24^ For the analyses, we defined deductive codes, which we developed and summarized in a codebook based on findings from the same scoping review that informed the interview guide (see Appendix 2).^8^ After the first interview round (*n* = 8, Figure 1), we coded the interview texts with deductive and new inductive codes emerging from topics not yet covered in our codebook. The deductive and inductive codes were then grouped into overarching themes and subthemes. In the second round of interviews (*n* = 5, Figure 1), we coded the interview texts after each interview with the codes defined in the first round, along with new inductive codes emerging from topics not yet covered in the previous interview round. We then mapped these codes to existing and as well as new themes and subthemes. All the codes used in the first and second interview rounds informed the development of the final themes and subthemes of this study. We interviewed experts until thematic saturation was reached, which was identified by the completeness and replicative nature of the data after 13 interviews.^25^

All interviews were conducted by one author (PD), and another author (VN) assessed the comprehensiveness of the codes, as well as their alignment to the themes and subthemes generated in the two interview rounds. Interviews were transcribed using an artificial intelligence (AI)-based transcription software, Otter.ai (version 2.1.41.612). The text was further cleaned manually in case of typographical errors. The coding and analysis of the interview transcripts were done in MaxQDA (version 10), a qualitative data analysis software.^26^

## Results

### Participant demographics

We invited 38 principal investigators, researchers, and software developers, 13 (13/38, 34%) of which were finally enrolled. Interviews were conducted between April and May 2022. Most participants are researchers (11/13, 85%), around half of which are principal investigators in recent remote digital health studies (6/11, 55%). Two interviewees (2/13, 15%) are software developers for digital technologies used in recent remote digital health studies, one (1/2, 50%) of whom is also a researcher. Most participants are based in the United States (6/13, 46%) or the United Kingdom (6/13, 46%), while one participant (1/13, 7.7%) is based in France. The average interview length was 62 minutes.

### Themes

We identified four major themes related to increasing participation in remote digital health studies: (1) study planning to increase participation; (2) participant enrollment; (3) participant retention; and (4) future requirements for study planning. Subthemes were assigned to each major theme, all of which were deductively mapped to the UTAUT constructs: (a) performance expectancy, (b) effort expectancy, (c) social influence, and (d) facilitating conditions (Appendix 3).^23^

#### Theme 1: Study planning to increase participation

##### Sub-theme 1a: Identifying participation motivation factors

The interviewees stressed that researchers should understand the motivations that the participants have to enroll in a study. For example, in the context of chronic conditions, the interviewees found that partnering with a clinical site and getting a clinician's referral, as well as appealing to the participants’ need to understand and manage their conditions were crucial motivators to participate.

*Lots of people, believe me, they sleep badly. They believe it affects their quality of life and they feel like no one [believes them]. So they’re interested in answering it.* (researcher)

When recruiting healthy participants, the interviewees found that appealing to altruism often facilitates participation. An example mentioned by many interviewees was the observed increase in study participation during the COVID-19 pandemic. They emphasized that during this time, many participants were driven mainly by altruistic motives, as well as the longing to connect to something wider.

Compared to traditional studies, the interviewees emphasized the additional complexities that come with defining participants’ motivations to join digital health studies. They highlighted that these complexities require an understanding of participant motivations at very early study stages. Involving participants early in the study design, such as through patient and public involvement (PPI), may allow researchers to best adapt study design and outreach.

*If someone's committing to a clinical trial, the ask for them is clear and there's a lot of reason for them to [participate]. Whereas in [remote] digital health [studies], the pressure is not there and [researchers] need to use different ways to get to that audience*. (software developer)

##### Sub-theme 1b: Identifying engagement and disengagement factors

The interviewees emphasized that remote digital health studies require additional efforts to engage the participants, regardless of whether they are highly motivated or not. Some mentioned that the (a) lack of in-person interactions and (b) abundance of health technologies often lead to a quick loss of interest and reduced engagement. Others underlined the importance of continuously observing participant responses to study tasks and promptly initiating follow-ups in cases of lower engagement. Understanding what engages or disengages the participants is crucial for that.

*[With remote digital health studies] you’re trying to stop [participants] from leaving. So, you look at what's the reason for disengaging and counter those. For example, if people are dropping out after the third questionnaire, because it's too long.* (software developer)

Once the engagement factors are known, the interviewees underlined that it is key to integrate them into the study design. For example, some mentioned the use of co-design or citizen science approaches to increase participants’ sense of ownership in a study. One interviewee emphasized the importance of continuous participant acknowledgment throughout the study conduct as one approach to engage the participants in the study.

*[Researchers should provide] at least a sense of ownership and partnership in the study. If you make [the study] so participant-centric in terms of having them feel a part of the study, it makes such a difference for the adherence.* (principal investigator)

#### Theme 2: Participant enrollment

##### Sub-theme 2a: Choosing the right recruitment strategy

Large-scale adverts were the most prominently reported approaches to recruit participants for remote digital health studies. The interviewees shared experiences with studies that rapidly went “viral” shortly after advertising on social media or within widely used apps (e.g. Fitbit). Some mentioned that choosing catchy study names and addressing questions that are relevant to the public or that people care about can boost enrollment.

*A large amount of people believe that the weather affects their pain. Lots of people wanted to take part in [the study] so that they could prove that it was right. It [also] got a lot of media attention [because] the name is very catchy.* (researcher)

Adverts through Facebook or Google Ads were described as too costly. As alternatives, the interviewees suggested multi-channel recruitment strategies, advertising at events, or through part-time job portals. The interviewees also reported high enrollment in studies where participants’ altruism was targeted through advertising, such as by highlighting the impact of participation on advancing research for a disease.

*[We advertised the study on Craigslist] as a part time job, not volunteering, not research. It was the cheapest, and it was the best one. I feel it was kind of a side entry into getting people interested in doing this stuff.* (principal investigator)

Related to large-scale advertising, the interviewees warned that there is a relatively high risk of recruiting unrepresentative samples. For more targeted recruiting efforts, some suggested hiring external recruitment companies or recruiting participants directly through patient advocacy groups, charity organizations, and clinical sites.

##### Sub-theme 2b: Assisting participants during enrollment

The interviewees generally agreed that additional assistance and communication efforts are necessary in remote digital health studies. These were often linked to the further measures required to enroll and onboard participants into a study to create a seamless experience.

*When the [clinical] trial is essentially on their smartphone, or they’re wearing a trial on their wrist, I think there's an obligation from the research team that we be available 24/7 for bi-directional communication.* (principal investigator)

One of the proposed measures was the availability of staff to support with onboarding. This can be in the form of providing understandable written and audiovisual information as well as offering the participants the option to directly talk with the study team in case of arising problems. If the participants seek support, the interviewees stressed the need for researchers to be patient and supportive, especially in studies involving older participants, to avoid discouraging their participation due to technology-related challenges.

*During onboarding we've always acted sort of as the interim tech help desk, with the app provider [helping us] answer the properly technical questions. You also want [participants] to have as much material as possible to do [this] on their own, but they need to be able to get in contact with you and not feel rushed.* (researcher)

##### Sub-theme 2c: Promoting inclusivity

Many interviewees identified a lack of trust in healthcare and research as a critical inhibitor to reaching and enrolling marginalized and underserved populations. To achieve inclusivity, the interviewees stressed the importance of identifying those who are not yet systematically included in remote digital health studies. They emphasized that this process should not be limited to ethnic diversity, but also focus on cultural subcommunities and stigmatized populations (e.g. individuals with mental health disorders). Interviewees reported partnering with patient advocacy groups as a critical step in fostering trust within these communities.

*Know your target community first. If literature suggests that a certain community is suffering more from chronic pain, start from there. Identify specific groups and then ensure the additional resources you need to target them.* (researcher)

The interviewees emphasized the critical role of community members in co-designing study elements that foster inclusivity. Co-design can inform the ways a study enrolls (and reaches) participants, making it more inclusive and appropriate. Using targeted audio/visual material, translated text, and adapted wording and formatting based on regional testing are some of the mentioned approaches informed by co-design and key to more inclusive remote digital health studies.

*Through user testing, the adaptations we’ve made [for an app for Parkison's patients] included making sure the contrast is high, [increasing] the size of the text, [adjusting] the tone of voice, and [adapting] the wording by working with clinicians and patients. You have to do regional testing as well. It's not just translation.* (software developer)

#### Theme 3: Participant retention

##### Sub-theme 3a: Providing incentives

Overall, engaging participants in managing their health conditions was described as a key incentive to study participation. The interviewees also emphasized that researchers should provide information back to participants. Examples included creating reports or dashboards where participants can access and share their data. Allowing participants to opt-in or opt-out of receiving their data was described as essential, especially when considering the context of disease progression. The interviewees also found that communicating study outcomes to participants at the end of studies often led to subsequent participation in future studies.

*There's a really interesting self-management aspect where [the user] understands [herself]. And it's very simple, you just collect symptoms in a really nice way [and] give some sort of visualization back, [which] creates really good engagement.* (software developer)

Many interviewees found that financial compensation contributed to fast task completion and long-term engagement. However, some interviewees found that offering more financial compensation does not always result in improved retention. The interviewees also emphasized that financial incentives alone are not enough for engagement. On the contrary, financial incentives can also attract bad actors who attempt to cash in financial compensation without participating in the study. As safeguards, the interviewees suggested recruiting participants directly through health centers or patient advocacy groups. Other approaches included screening questions or not advertising financial compensation during enrollment.

*We ended up stopping the study and put in a number of rules to screen out bad actors. Some of them [were screened out if they had] IP addresses outside of the US, or if they had other behaviors, like if passively synced data never showed that the phone actually moved, [which points to] cell phone farms.* (principal investigator)

However, all interviewees agreed that the definition of incentives goes beyond financial compensation. Multiple interviewees mentioned that creating within-study online communities can act as an incentive and increase study retention, allowing participants to interact with each other (or online coaches) and exchange their experiences.

*Adherence was almost 100%. Everybody felt a humongous sense of connection. There was like a mini like Facebook amongst the participants, where they would post photos and people liked them. And there was a pop from knowing that there was a coach in the background and that they had regular check ins with.* (principal investigator)

Finally, when asked about gamification incentives, the interviewees agreed that gamification is often tokenistic and may feel patronizing to participants. Some found that it only worked in specific contexts, such as with children, or rewarding participants by feeding data back to them.

*You've got to be careful you're not creating a tokenistic [gamification] system that works very short term. You have to use it in the right context. For certain types of engaged individuals it makes sense, such as cyclists, who are motivated and competitive. But that's not your typical user type for research.* (software developer)

##### Sub-theme 3b: Communicating with participants

The interviewees also emphasized the benefit of developing a rapport with their participants, which they can do by sending check-in messages, answering emails promptly, and acting as a friendly contact rather than a clinical one. For longer studies, the interviewees suggested automating contact as much as possible, such as through timed check-in messages.

*Regular check ins, little things like emails [where we say] ‘hey, we’re here’ go a long way. We found that some element of back and forth is the most important thing. It's not just like an Android robot running in the background.* (principal investigator)

The interviewees mentioned that sending reminders with strategic timing and wording can help with participant engagement. For example, sending more frequent reminders before important and more intensive study tasks (e.g. longer surveys), or timing reminders to be sent after participants have been inactive for certain periods of time (e.g. longer breaks). The interviewees also suggested game or loss framing of messages and sending automated motivational reminders with a human touch, for example, every 5 days.

*We’ve sent [automated] text messages as if a human's actually written them. If it's a comma in the wrong place, that's how I would write the message. We’ve had people contact us to say, ‘thank you for taking the time to send me a message.’ It makes people feel valued. This is about someone contributing with the respect that they deserve.* (researcher)

Lastly, the interviewees highlighted the benefit of sending participants messages on a semi-frequent basis to communicate how valuable their contribution to the study is, such as through newsletters, social media, or a handwritten postcard sent to their homes.

##### Sub-theme 3c: Minimizing effort and complexity

The effort and complexity required to complete a study were described as barriers to participant retention. The interviewees provided some examples on how to mitigate these: first, by allowing participants to personalize the timing by which they complete study tasks (e.g. surveys) to fit better into their daily lives; second, if possible, by combining low-burden passive data collection (e.g. sensors) with routine active data collection initiated by participants (e.g. surveys); third, whenever appropriate, pairing data collection with self-management (e.g. ad hoc symptom tracking), making the process more meaningful and valuable to participants; and fourth, by being transparent and informing participants a-priori about the length or complexity of an upcoming study task.

*If you can get someone into a quick daily routine of filling in their data, you’ll get it to stick. Once it drops off and it's weekly, it gets harder. [Data collection tasks must] be in the right area and useful to the individuals. They’ve got to see the benefit.* (software developer)

Choosing the right frequency and length of data collection to minimize participant burden was described as a challenging task, especially because the appropriateness of data collection methods ultimately depends on many factors (e.g. health characteristics of the target population, research question). The interviewees recommend testing the associated tasks or surveys iteratively with the target groups. This can help recognize if certain tasks may be too burdensome for some populations, such as individuals living with certain chronic conditions. Furthermore, the interviewees suggested having all the enrollment and study steps, such as registering an app or connecting a device, on one platform to reduce burden.

##### Sub-theme 3d: Choosing the right technology

The interviewees highlighted that technology should be used to support a study rather than conduct it, emphasizing that researcher involvement is necessary during most study steps. The interviewees found that a help desk should be available to support participants during technology setup. The interviewees also reported positive feedback from studies where all study steps were in one platform and customizable (e.g. messaging preferences).

The interviewees also emphasized that technologies work best when adapted to the target population and the study's research aims. This can be done, for example, by developing a smartphone app with custom designs and assessments specifically tailored for one study. However, this is an expensive endeavor and often not feasible. Using platforms that provide ad hoc customizable software solutions was described as a cost-efficient alternative. Overall, the interviewees stressed the positive impact of a seamless user experience on study engagement.

*For remote digital studies, you must have that consumer level of frictionless experience. [With] apps in the App Store, [no one is] holding your hand, telling you “Press this button.” It's got to be intuitive. Otherwise, people will just go, “I’m out.”* (software developer)

The interviewees' experiences with chatbots were mainly negative, based on observations that they failed to mimic a human connection. The interviewees suggested alternative cost-effective solutions, such as the Apache Kafka messaging framework, which adapts the delivery of study tasks in a way that is more relevant to the participants' context.

*You can take the data you’re getting in real-time, analyze it and do something with it. That might be [noticing you] had a bad night's sleep and sending a questionnaire about sleep. You want to give that piece of information at the point of context.* (software developer and researcher)

#### Theme 4: Future requirements for study planning

##### Sub-theme 4a: Adapting the scientific requirements

Based on their experiences, the interviewees highlighted that future remote digital health studies required some adaptations. First, since many are observational and lack the engaging interventional components of traditional trials, overrecruiting might be essential. They emphasized that overrecruiting or calculating target sample sizes should be based on assumptions about the target population under intention-to-treat or per-protocol principles. For example, in a study that targeted only healthy participants, researchers overrecruited based on an expected higher dropout rate.

There were also reports of autocorrelation from the large amount of individual-level data collected in remote digital health studies over prolonged periods. To counteract this, the interviewees suggested reporting expected effect sizes with certain types of autocorrelation in study protocols or adjustments using statistical methods. The interviewees also suggested matching self-reported data with data from sensors to ensure higher data quality. Finally, some interviewees suggested running pilot trials to make sure that everything related to data collection and interaction points with participants runs smoothly.

##### Sub-theme 4b: Fostering guidelines and innovation

The interviewees were optimistic about the future of remote digital health studies involving high participation. However, some mentioned that, for now, strategies to increase participation are not being implemented in a systematic and evidence-based way. Guidelines to inform study planning and design, with an emphasis on successful participation were reported to be important future steps. Sharing best practices will be an essential step toward that.

*Many [researchers] are still shooting from the hip, which means they don’t know the principal approaches on, for example, how to consider differences in retention. Most people go, ‘That's not a problem’ because they don’t know it's a problem.* (researcher)

Finally, the interviewees emphasized the importance of developing innovative approaches to advance and support participation in remote digital health studies. One of these is AI to automatically adapt communication with participants or providing real-time financial incentives based on participant engagement. An interviewee also suggested the benefit of planning and conducting studies with a goal set to learn from every aspect of the study. Emphasis was also placed on the need to continuously adapt research protocols, in line with emerging participant, technological and societal requirements.

*I’m a big believer in this concept of ‘complexity science’ in that researchers learn from every aspect of a study. We need to learn if outreach is working, if people fill out the survey and then stopping at this point, [and the reasons why]. We need to re-evaluate and iterate on it and saying “Okay, what's working? What isn’t?”* (principal investigator)

## Discussion

We identified four themes relevant to successful participation in remote digital health studies: (1) study planning to increase participation, (2) participant enrollment, (3) participant retention, and (4) future requirements for study planning. Facilitated by the UTAUT framework,^23^ these themes reveal the breadth and interrelated aspects associated with planning as well as conducting such studies to increase participation. This study confirms and expands on the results of our previous work on factors that enable participation in remote digital health studies.^8^

### Digital and person-centered

Our findings underscore the need to integrate human elements in study planning and conduct to address the absence of in-person interactions in remote digital health studies. This is highlighted in the UTAUT analysis, with the “social influence” construct appearing across the person-centered study planning and participant enrollment themes. The literature also supports this observation, with studies suggesting that approaches, such as personalized motivational messages^27^ or online community involvement,^3^ could increase participation. However, experts suggest that these may not be successful without a comprehensive exploration of participants’ motivations and preferences prior to study planning, such as through PPI workshops and co-designing of digital health tools. Recent literature reinforces this, emphasizing the involvement of advocacy groups and citizen networks as well, to inform the development of digital health tools, study design, and participant outreach.^28,29^ Such efforts also aid in uncovering barriers to participation in remote digital health studies. Experts noted that waning study participation could partly stem from the proliferation of digital health tools, echoing literature's ambivalent view on their use in research, as facilitators or as barriers.^30,31^ Technology promises wider reach, easier access and more flexibility, all of which can incentivize study participation. Yet, anything less than a frictionless user experience can deter study participation of specific population groups, like older adults or other vulnerable, underrepresented populations, who may perceive technology use as challenging.^32,33^ Developing seamless technologies can be very costly and needs to be accounted for when planning a remote digital health study.^34,35^

### Measures beyond technology

While technology is there to support, the role of researchers remains essential. This notion becomes evident with the UTAUT analysis, whereby the “facilitating conditions” construct is present across all subthemes in the participant retention theme. Generally, experts noted that participants tend to expect continuous researcher availability for regular interaction throughout the study. This is aligned with recent studies that highlight participants’ expectations around communication with researchers, such as for technical assistance.^31,36^ In what form and how researchers are present ultimately depends on the study's setup (e.g. communication channels), size, and budget limitations.^37^ A proposed approach was working with an external research marketing firm. However, researchers must be cautious about potential participant harassment from constant communication efforts during the study.^38^ In study phases with expected high volumes of communication, such as during study enrollment, experts advise having a researcher team available that answers emails promptly and provides support in case of any issues. Equally as important is the role of incentives. Providing financial compensation is arguably the most widely-implemented incentive in remote digital health studies. While numerous studies attribute financial incentives as the main drivers of participation in their studies,^7,39,40^ our findings highlight that financial incentives alone might not be enough to ensure adequate study participation. Meaningfully engaging participants, while providing them feedback and access to their own data, was described as additional and very important incentives, which are scarcely reported in the literature.^8^

Furthermore, researchers need to implement additional measures to include participants in remote digital health studies who have previously been systematically excluded. Common strategies mentioned in the literature involve adapting enrollment processes, such as by offering translated materials for specific cultural groups, and adjusting study structure and duration to reduce participant burden.^41–43^ However, an expert stressed that an often neglected first step in study planning is accurately identifying relevant marginalized subcommunities, as well as their barriers to participate in remote digital health research. The role of researchers to identify and include these population groups is essential, such as by involving target community research staff for communication and co-developing material delivery with community members. Yet, among the experts we interviewed, only three had direct experience with remote digital health studies focused on promoting inclusivity, underscoring the early stages of this matter.^44^

### Recommendations and future guidance

The summarized recommendations for study planning and conduct to promote participation in remote digital health studies are presented in **Textbox 1**.

**Text Box 1.** Recommendations for participation in remote digital health studies based on the study's findings.

**1) Study planning to increase participation** – Identify the motivation factors for participants to enroll in remote digital health studies. – Identify the factors that engage or disengage participants during remote digital health study conduct.
**2) Participant enrollment** – Choose the appropriate recruitment strategy based on the target population and study goals. – Provide assistance to participants during study enrollment, in the form of personal and technical support for enrollment study tasks. – Take additional precautions to accommodate cultural subcommunities and stigmatized populations in remote digital health studies.
**3) Participant retention** – Provide incentives based on participant and study factors that go beyond financial compensation. – Communicate regularly with participants, especially in phases of the study with high task loads. – Deliver the key study steps in a way that minimizes participant burden and task complexity. – Adapt the choice of technology to facilitate the delivery of the study.
**4) Future requirements for study planning** – Align the study's scientific requirements with measured expectations of study participation success. Share best practices and foster innovation to support high participation in remote digital health studies.

This study's analysis and resulting recommendations were shaped by the UTAUT framework.^23^ Overall, we found the UTAUT framework to be highly adaptable to our study, as evidenced by its comprehensive coverage of all UTAUT constructs across the various themes identified in the analysis. In particular, the findings from the UTAUT analysis underscored the important role of researcher involvement and tailoring of digital health tools to meet participant needs, as well as to reduce their burden. Our study's recommendations also expand on current standards, such as mERA^45^ and CONSORT-EHEALTH,^46^ which primarily address the methodological aspects of integrating technologies in traditional study settings, by offering approaches to reach and retain participants in ways that are inclusive, engaging, and beneficial. Overall, our study's findings and preliminary recommendations present an invaluable opportunity to develop more awareness and outlets for sharing experiences in a structured format in future research or a symposium with experts.

## Limitations

This study's findings provide a wide breadth of approaches that can inform strategies to increase participation in remote digital health studies from various experts. However, our study presents some limitations. Across all interviews, there were few findings that were agreed upon by all interviewees. We attempted to mitigate this by implementing a second sampling round, where we recruited participants until we achieved thematic saturation. Nevertheless, the qualitative nature of this study prevents our findings to be representative for all remote digital health studies. Furthermore, our study's focus on recruiting experts from published work may have omitted findings from the field that have not been published yet. Lastly, using personal contacts through snowball sampling may have added a subjective component to our findings.

## Conclusion

Remote digital health studies present an invaluable opportunity to conduct research with large, diverse, and representative study samples. However, ensuring that participants are enrolled and retained until study completion remains challenging. To promote participation, experts suggest that (1) remote digital health studies are planned on the basis of adequate knowledge of what motivates, engages, and disengages a target population, (2) enrollment strategies are selected carefully, attached to adequate support, and focused on inclusivity, (3) retention strategies minimize the effort and complexity of study tasks and ensure that technology is adapted and responsive to participant needs, (4) experiences and innovative approaches for participation are actively shared. Ultimately, these efforts are resource-intensive and challenging to implement. While technology could enhance scalability, there is little alignment between human and technological aspects in remote digital health studies. In future remote digital health studies, experience-based guidance can shape study planning and guidelines for a balanced approach to achieve personalized, innovative, and scalable study designs with researcher involvement to enhance participation.

## Supplemental Material

sj-docx-1-dhj-10.1177_20552076231212063 - Supplemental material for Promoting participation in remote digital health studies: An expert interview study Click here for additional data file. Supplemental material, sj-docx-1-dhj-10.1177_20552076231212063 for Promoting participation in remote digital health studies: An expert interview study by Paola Daniore, Vasileios Nittas, Felix Gille and Viktor von Wyl in DIGITAL HEALTH
