# Supplementary material for: Promoting participation in remote digital health studies: An expert interview study
Source: Digit Health. 2023 Nov 13;9:20552076231212063. doi: 10.1177/20552076231212063 (PMC10644759; doi:10.1177/20552076231212063)
Supplement: sj-docx-1-dhj-10.1177_20552076231212063 - Supplemental material for Promoting participation in remote digital health studies: An expert interview study [file sj-docx-1-dhj-10.1177_20552076231212063.docx]

**Appendix 1**. *Interview guide, informed by the UTAUT framework and scoping review on participation in remote digital health studies*

**Introductory questions**

1. Tell me a bit about yourself, your work and professional background.
2. Tell me a bit about your experiences as a researcher in digital health and working with remote cohorts in your current research and/or previous studies.
3. What are the general key factors you believe enable a successful digital health study with remote cohorts?

**Participant motivation profiles and provision of incentives**^1^, ***performance expectancy***^2^

1. What are the key factors you believe enable high study enrollment with remote cohorts?
2. What are the key factors you believe enable high study completion with remote cohorts?
3. What are the key factors you consider when planning a digital health study with remote cohorts from a participant engagement perspective (e.g., provision of incentives)?
4. [**If answered “yes” to question 6**] Please elaborate on additional provisions you have taken (e.g., provide incentives) to enable higher participation in digital health studies with remote cohorts. Did you receive any feedback from the participants?
5. [**If answered “yes” to question 6**] Was a there a specific choice on the timing in which these provisions were made available during the study (e.g., incentives provided at recruitment vs. end of study)? What motivated these choices (e.g., theory, literature)?
6. In previous or current remote cohorts, do you aim to recruit specific participant profiles? How do you make these choices?
7. Have you encountered participants who entered studies for financial gain and drop out (“gaming”) after receiving the incentives? If so, did you take any mitigation strategies again these kinds of participants?

**Complexity of tasks required from participants**^1^, ***effort expectancy***^2^

1. Was a there a specific reasoning behind the choice of tasks asked from the participants (e.g., number of tasks/questions/length of questions)? What motivated these choices (e.g., theory, literature)?
2. Did you encounter high rates of attrition in any of your previous studies with remote cohorts? If so, what measures did you take to reduce attrition in future studies (or to retain cohorts)?
3. What motivated the choice of technologies (or analytical tools) used in your current or previous studies? Did you receive any feedback from the participants on the ease-of-use of such technologies (or feedback on the analytical tools)?
4. Did you by any chance adapt the choice of technologies / analytical tools used in the study to enable higher participation?

***Social influence***^2^

1. Which recruitment approaches worked best in your experience? Did you find that, in your previous or current studies, your recruitment strategies attracted certain population groups (e.g., race, age, gender)?
2. Did you make additional efforts to recruit marginalized populations in your remote cohorts? If so, what efforts did you make? Did you encounter any issues?
3. Did you find that certain population groups were more likely to be retained longer in a study (e.g., race, age, gender)?
4. Did you study involve any AI/ML algorithms in your studies? Was the use of such algorithms made transparent to participants before their involvement in the studies? How do you believe this may have impacted participation in the study?

***Facilitating conditions***^2^

1. Have you adapted methodologies of previous digital health studies with remote cohorts to enable higher participation / participant engagement (e.g., provided assistance)?
2. Did you encounter any issues with varying levels of digital literacy in working with remote cohorts? Did you take any measures to address these issues? Did you adapt parts of your methodologies to accommodate these participants (e.g., onboarding sessions)?
3. What retention strategies worked best in your experience? Did you make additional efforts to retain marginalized populations in your remote cohorts? If so, what efforts did you make? Did you encounter any issues?

**Scientific requirements of the study**^1^

1. What are the key factors you consider when planning a digital health study with remote cohorts from a scientific requirements perspective (e.g., sample size)? Do you refer to guidelines or frameworks to help the planning process?
2. [**Possible follow-up to question 22**] How has study planning with remote cohorts differed methodologically from planning “traditional” studies without remote components?
3. What are your future requirements in enabling higher participation in digital health studies (surveys) with remote cohorts from a study planning perspective?
4. What tools and resources (e.g., guidelines, frameworks) do you believe are still missing but could be beneficial to enable higher participation in digital health studies (surveys) with remote cohorts?
5. What is your vision for digital health studies (surveys) with remote cohorts in the future studies?

**Appendix 2**. *Codebook*

| **Factors that affect participation**^1^ | **Definition** |
| --- | --- |
| Participant profiles | The demographics of participants who join a remote digital health study can differ based on personal motivations, the question the study wants to answer, as well as various other factors. Planning of remote digital health studies can benefit from understanding the requirements of these subpopulations and adapting their methods to include a more diverse sample in their study. |
| Participants’ motivation to participate in study | The motivations for participants to join a remote digital health study can be classified as intrinsic or extrinsic. Intrinsic motivation to participate in a study may be motivated by altruistic motives or by hopes for medical advances, especially among individuals with chronic diseases. Extrinsic participation can be motivated, among others, by investigators in the form of monetary incentives or clinical referrals. Understanding of participants’ motivations to participate in a remote digital health study can inform study planning to adapt the study’s methodology accordingly. |
| Provision of incentives | The provision of incentives or nudges may help achieve higher remote digital health study enrollment and retention. To do so, study planning should consider the research contexts in which incentives or nudges should be provided, with a focus on the participation profiles, their motivations and the study’s requirements. Planning of remote digital health studies can also benefit from considering the timing in which incentives should be provided to enable higher participation. |
| Gaming of incentives | The provision of incentives may attract bad actors who enroll in a study to collect financial incentives and then drop out. Remote digital health studies that plan to provide incentives should take these subpopulations into account in their study planning by e.g., adding a gatekeeping step during study enrollment. |
| Provision of assistance | Provision of assistance during the study may improve digital literacy skills of participants, while also instilling a sense of trust between the participant and the researcher through personal interactions. Remote digital health study planning should consider the required additional resource requirements to be able to assist large remote cohorts. |
| Complexity of tasks | Typical tasks required of participants include physical activity tasks, such as walking a certain number of steps every day, as well as essential tasks for the study, such as signing an informed consent form, as well as mental tasks, such as the measure of cognitive burden of a participant. The complexity of tasks required from the participants, along with the expected duration of the study, can impact study enrollment completion. |
| Scientific requirements of the study | Scientific requirements define the study design and expected target sample size. Therefore, scientific requirements set the goal of the study, while task complexity or incentives or nudges enable study goal achievement. Consideration of participant requirements in remote digital health study planning may help increase the statistical power and reduce selection bias of the study. |

**Appendix 3**. *Themes from qualitative interviews mapped to the Unified Theory of Acceptance and Use of Technology (UTAUT) framework*

**References**

1. Daniore P, Nittas V, von Wyl V. Enrollment and Retention of Participants in Remote Digital Health Studies: Scoping Review and Framework Proposal. *J Med Internet Res*. 2022;24(9):e39910. doi:10.2196/39910

2. Chiu TM, Eysenbach G. Stages of use: consideration, initiation, utilization, and outcomes of an internet-mediated intervention. *BMC Med Inform Decis Mak*. 2010;10(1):73. doi:10.1186/1472-6947-10-73
